# High Temperature Optical Fiber Sensor Based on Compact Fattened Long-Period Fiber Gratings

**DOI:** 10.3390/s130303028

**Published:** 2013-03-04

**Authors:** Ruth I. Mata-Chávez, Alejandro Martínez-Rios, Julián M. Estudillo-Ayala, Everardo Vargas-Rodríguez, Roberto Rojas-Laguna, Juan C. Hernández-García, Ana D. Guzmán-Chávez, David Claudio-González, Eduardo Huerta-Mascotte

**Affiliations:** 1 Espintrónica y Óptica Aplicada-CA, Departamento de Estudios Multidisciplinarios, DICIS, Universidad de Guanajuato, Av. Universidad s/n, Col. Yacatitas, Yuriria, Gto, 38940, Mexico; E-Mails: evr@ugto.mx (E.V.-R.); ad.guzman@ugto.mx (A.D.G.-C.); dclaudio@ugto.mx (D.C.-G.); eduardoh9@hotmail.com (E.H.-M.); 2 Centro de Investigaciones en Óptica, Loma del Bosque 115, Col. Lomas del Campestre, León, Guanajuato, 37150, Mexico; E-Mail: amr6@cio.mx; 3 Optoelectrónica-CA, Departamento de Electrónica, DICIS, Universidad de Guanajuato, Carr. Salamanca-Valle Km 3.5 + 1.8, Palo Blanco, Salamanca, Gto, 36855, Mexico; E-Mails: julian@ugto.mx (J.M.E.-A.); rlaguna@ugto.mx (R.R.-L.); jchernandez@ugto.mx (J.C.H.-G.)

**Keywords:** long-period fiber grating, temperature sensor, electric arc, dispersion shifted fiber, fattening, 42.81.-i

## Abstract

A compact high temperature fiber sensor where the sensor head consists of a short fattened long period fiber grating (F-LPFG) of at least 2 mm in length and background loss of −5 dBm is reported. On purpose two different F-LPFGs were used to measure temperature variations, taking advantage of their broad spectrum and the slope characteristics of the erbium light source. This approach affected the spectrum gain as the linear band shifting took place. The measured sensitivity of the long period fiber gratings were about 72 pm/°C in a range from 25 to 500 °C. Here, the temperature rate of the experiment was 0.17 °C/s and the temperature response time was within 3 s. Moreover, temperature changes were detected with an InGaAs photodetector, where a sensitivity of 0.05 mV/°C was achieved.

## 1. Introduction

Optical fiber devices compete with conventional mechanic and electronic sensors due to their physical advantages such as compact size, light weight, immunity to electromagnetic interference, high sensitivity and temperature resistance [1–3]. There is a simple and low cost fabrication procedure to fabricate fiber gratings, the arc discharge method, which is applied to any type of fiber using a fusion splicer machine. The standard fabrication method varies the quantity of tension applied over the fiber and leads to tapered gratings [4,5]. Theory and functionality of these optical devices is completely known, as well as their sensitivities, which depend on fiber type, the grating period and the external perturbation [6,7]. These types of LPFG are well suited for many sensing applications but sometimes their length of more than 10 cm is a great disadvantage. Long period gratings in two and three layered optical fibers, as well as photonic crystal LPFG had been proposed for temperature measurements using electric discharges [8–13]. Studies of thermal behavior have been reported for LPFG with different core dopants, which were fabricated with electric arc applying the tension method and a period of 540 μm for up to 1,200 °C [14]. Rego *et al.* presented a study in LPFGs thermal behavior on different types of optical fibers, including Corning DSF with this method [15]. Sakata *et al.* proposed an intensity-based fiber sensing technique where temperature sensing was made by intensity detection by employing a combination of a light-emitting diode (LED) and a photodetector [16].In 2008, the *fattening* method was proposed, as an alternative method, to fabricate F-LPFG.A commercial fusion splicer machine and a dispersion shifted fiber were used and approximately3 mm in length gratings were achieved [17]. A F-LPFG is a LPFG formed by enlarging the fiber diameter applying several arc discharges in the same region. Periods of minimum of 100 nm can be applied since there is a geometrical contribution induced through the fattening or compression of the fused sections which changes the dimensions of the fiber structure, *i.e.*, the core and cladding dimensions will increase. This geometrical change will modify the effective indices of the interacting modes as in the case of tapered fiber where the geometrical change affects the wave-guiding properties.

We present an enhanced temperature characterization in short F-LPFGs for sensing implementation comparing with results of our previous work. This is, a spectral shift of ∼50 pm/°C at a temperature range from 25 °C to 190 °C was obtained with fattened positions separated by ∼25 nm, where at each position the fusion process is repeated five times resulting in a total overlap distance of ∼55 μm. A low power white light source was used [17]. In this work two individual F-LPFGs with 1,524 and 1,546 nm central dip wavelengths were fabricated. Temperature variations were characterized in transmission using an EDF light source as the excitation source. Two different methods of detection for the output were used, an OSA and InGaAs photodetector. The measuring range of temperature was of 25 to500 °C and the sensitivities calculated by each method were of 72 pm/°C and 0.05 mV/°C, respectively. Using advantageously the slope characteristics of the fluorescence spectrum of erbium, optional temperature measurements can be realized with these LPFGs.

## 2. Principle of the Fattened-Long Period Fiber Grating

An important parameter of a long-period fiber grating is known as the resonance wavelength and occurs when the phase matching condition between the core and the *m*-th cladding mode is satisfied. This condition can be written as:

(1) $$\lambda_{\text{res}}=\left\lfloor n_{\text{coe}}(\lambda_{\upsilon })-n_{\text{cle}}^{v}(\lambda_{\upsilon })\right\rfloor \Lambda$$

where Λ is the grating period, υ is the mode number and *λ_res_* is the resonance wavelength of υ-th order. *n_coe_*(*λ_υ_*) and $\eta_{\text{cle}}^{\nu }(\lambda_{\upsilon })$ are the effective indices of the core mode and the cladding mode, respectively. The modal effective index can be calculated by solving the characteristic equation obtained by considering a four-layered waveguide as the dispersion shifted fiber [17]. As temperature LPFG sensors, the temperature sensitivity of the resonance wavelength can be expressed as [11]:

(2) $$\frac{d\lambda_{v}}{dT}≅(C_{co}-C_{cl})(\eta_{01}-\eta_{0m})\Lambda \gamma$$

with:

$$\gamma =\frac{1}{1-\Lambda (\frac{dN_{01}}{d\lambda }-\frac{dN_{0m}}{d\lambda })}$$

where *η*_01_ and *η*_0_*_m_* denote the fractional powers in the core for the *LP*_01_ and *LP*_0_*_m_* modes. *γ* describes the effect of the modal dispersion evaluated at the resonance wavelength, and *C_co_*, *C_cl_*, *N*_01_ and *N*_0_*_m_* are the thermo-optic coefficients and the effective indexes of core and cladding, respectively [9].

**Figure 1.**
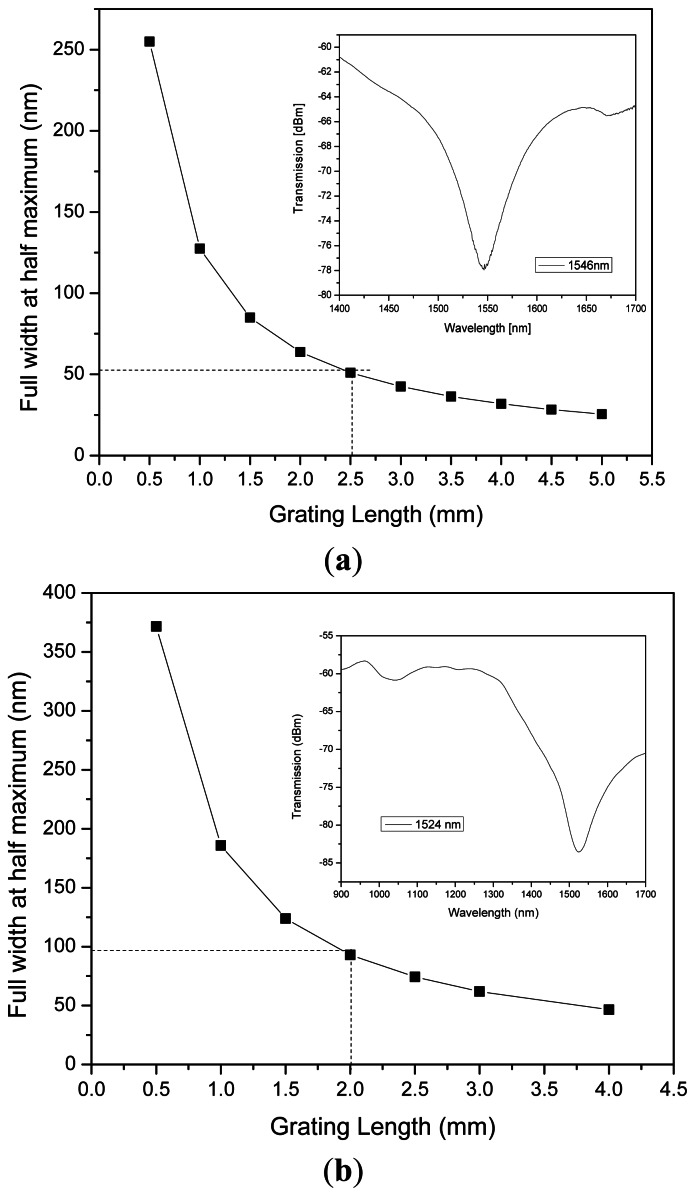
(**a**) Influence of the size of the F-LPFG with the dip bandwidth. Inset shows a 1,546 nm F-LPFG spectra al FWHM of about 51 nm. (**b**) A F-LPFG around 2 mm in length at 1,524 nm with a FWHM spectra of 90.43 nm.

The small size of the fattened grating contributes to the size of the bandwidth of the stopband measured at full width at half maximum [6], this is:

(3) $$\Delta \lambda =\frac{0.8\lambda^{2}}{L(n_{\text{eff}}-n_{cl})}$$

where *λ* is the resonance wavelength, *n_eff_* is the effective index of the guided *LP*_01_ mode, *L* is the grating length and *n_cl_* is the cladding refractive index. Figure 1 shows the bandwidth variation in aF-LPFG at 1,546 nm with its length. Shorter length gratings show broad spectra with higher slopes in the tails of the resonant band. Launching conditions do not change with temperature, so it is possible to measure these variations as intensity modulation with a wavelength laser source slightly off the resonance central wavelength.

The graphs were numerically obtained from Equation (3). The assumed parameters were estimated for a DSF using the BPM RSoft^®^ (Ossining, NY, USA) which results were based on the radii values:a = 2.9 μm, b = 5 μm and c = 8 μm, and the refractive index values n_1_ = 1.4598, n_2_ = 1.4498, n_3_ = 1.4498 and n_4_ = 1 of which the effective indices were obtained for different wavelengths.Of course these values might change for different types of fiber structures and refractive indexes so it is not an accurate indicator for LPFG lengths but illustrates the approximated size of the stopband measured at full width at half maximum.

We have to take into account that the electric arc is a variable which depends on external surrounding factors as humidity, temperature and the metal electrodes deterioration. Using an average effective index as for example Δ*n_eff_* = 0.015 and fixing the optical wavelength, different graphics can be obtained to serve as a guide for bandwidths obtained for an approximated F-LPFG length.

## 3. Fattened Long-Period Grating Fabrication

**Figure 2.**
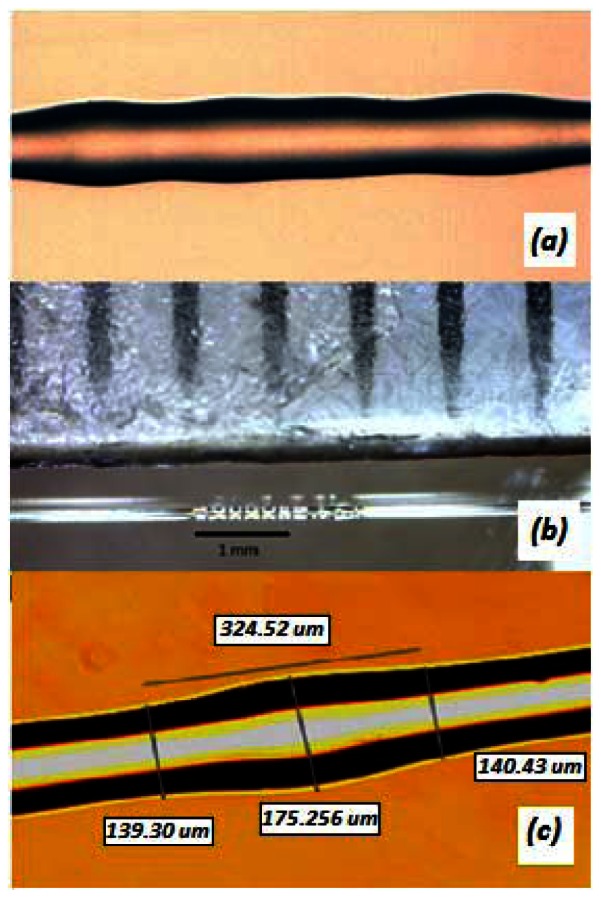
(**a**) Photograph of a F-LPFG in DS Fiber (**b**) F-LPFG with ∼2 mm length (**c**) DSF with fattened diameter with a maximum enlargement of ∼175 μm.

Using the electric discharge method with a fusion splicer machine (Fitel S175) in manual mode, the diameter of at least 2 mm length dispersion-shifted fiber (DSF) was enlarged by applying several electric arcs at the same point. The experimental setup for monitoring the evolution of the F-LPFG was a white light source coupled to one end of the DSF and the other end to the OSA. Splice discharge parameters that are mentioned in reference [16,17], were used to fabricate these particular gratings. The process was repeated five times along ∼2 mm of fiber length, until filtering functions were observed in the OSA [18]. Figure 2(a) shows a picture of the amplification of a fattened LPFG where the spaced glass lobes are observed and Figure 2(b) shows a scale in mm with the same F-LPFG of about 2 mm in length. Figure 2(c) shows that neither the lobes maintain the original diameter size of 125 μm nor the smallest diameter between the lobes.

The horizontal line represents the length of the fattened grating. This change in the whole fiber structure makes possible the coupling of modes to observe filtering functions at transmission spectra. Preliminary work has been done in order to find the fiber cladding modes for the F-LPFG. Using an ideal structure of the dispersion shifted fiber and the beam propagation method (BPM) of RSoft^®^ Software, the structure radii values were included as: a = 2.9 μm, b = 5 μm and c = 8 μm, as well as the refractive index values n_1_ = 1.4598, n_2_ = 1.4498, n_3_ = 1.4498 and n_4_ = 1 [18]. With this method we have found that the modes that are excited in the fiber grating, depending on the wavelength, are basically *LP*01 and *LP*11, as well as *LP*21 and *LP*03. We consider that these results are still not accurate due to the four layered structure. This is, the DSF has a core and a ring core with a depressed section between them and the rest is cladding. The analysis assumes an ideal single mode structure whose results cannot be said to be exact, but can be used as a guideline as a satisfactory method is found to obtain improved results.

This F-LPFG was fabricated with a grating period of 150 μm to achieve a central dip wavelength ∼1,546 nm. We must take into account the influence of the finite size of the electric arc, about 300 μm, over the affected surface area of the optical fiber. This makes it sometimes difficult to fabricate controlled central dip wavelength LPFGs with smaller grating period values. Although the strong geometric variation formed over the optical fiber structure makes possible to achieve a LPFG with a minimum of four grating periods. The F-LPFGs were obtained with a loss dip ∼15 dB, resonance wavelengths of 1,546 nm ± 20 nm and a broad bandwidth of more than 100 nm at full top width as observed in the inset of Figure 1.

Varying the number of arc discharges and alternating the grating period from 100 μm to 150 μm, a LPFG with a resonance wavelength at 1,524 nm was obtained. There is no accurate method to select the central wavelength in the fabrication process at this point, but we have observed that depends on the manufacturing process. Actually we work in the process optimization to reduce the background losses by fixing and enlarging the grating period and to find a form to select the exact location of the resonance band without losing its compactness. However, what we do is to change the program values as arc power, arc duration, and pre-fusion times as well as the number of arc discharges. That is, if we want to fabricate a F-LPFG around 1,550 nm, we apply five arc discharges with arc power of 85 mW, 650 ms for arc duration and 250 ms of pre-fusion time. After applying the five arc discharges the fiber is displaced 150 μm and start applying other five discharges. The obtained grating is about 3 mm in length with bandwidths around 55 nm. With this approach it has been possible to manufacture F-LPFG with central wavelengths around 1,300 nm near the cutoff wavelength, but it has been more difficult to manufacture them below the cutoff wavelength.

Opposed to the standard LPFG fabrication by electric discharge [15], this alternative method does not use a dead weight to apply axial tension over the optical fiber and it is not necessary to unscrew a fixture to place the fiber. An advantage over the UV method is that the grating is not erased or optically degraded with exposures to high temperatures (<200 °C). The hot push delay and the overlapping distance contribute to the grating formation and these values are advantageously established when manipulating the splicer machine in manual mode. Background losses can be reduced by applying arc discharges in manual mode which are of –5 dBm, and this is an advantage of the fabrication method. The fabrication process modifies the refractive index of the glass, giving the grating the filtering characteristics depicted in the inset of Figure 1(a,b). With the fattening method by electric arc, compact devices are always achieved and the advantage is that bulky and expensive equipment can be avoided for sensing interrogation, achieving in general, a whole compact sensing device. However, the fabrication of LPFG with electric arc is still not very accurate. Resonance wavelengths might result around ±5 nm for periods below 200 μm.

## 4. Fattened Long-Period Grating Characterization and Response

**Figure 3.**
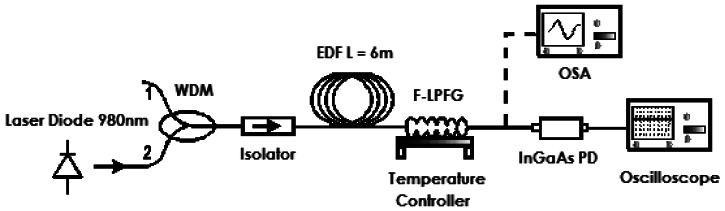
Sketch of the F-LPFG temperature sensor characterization system.

The experimental setup shown in Figure 3 was used to study the temperature effect over the transmission spectrum of the F-LPFG. The characterization system consists on a fluorescence light source, which is obtained by pumping an EDF with a laser diode (LD) at wavelength 980 nm through a wavelength division multiplexer (WDM 980/1,550 nm). An isolator was spliced to the common port of the WDM and the EDF which was employed to prevent any reflected light. Then the output of the source, another end of the EDF, was spliced to the F-LPFG (head sensor). The F-LPFG was put over a temperature controller with a range from 25 to 550 °C. Finally the output sensor could be coupled either to the OSA (spectral resolution 20 pm) or the InGaAs photodetector (PD)-oscilloscope.

**Figure 4.**
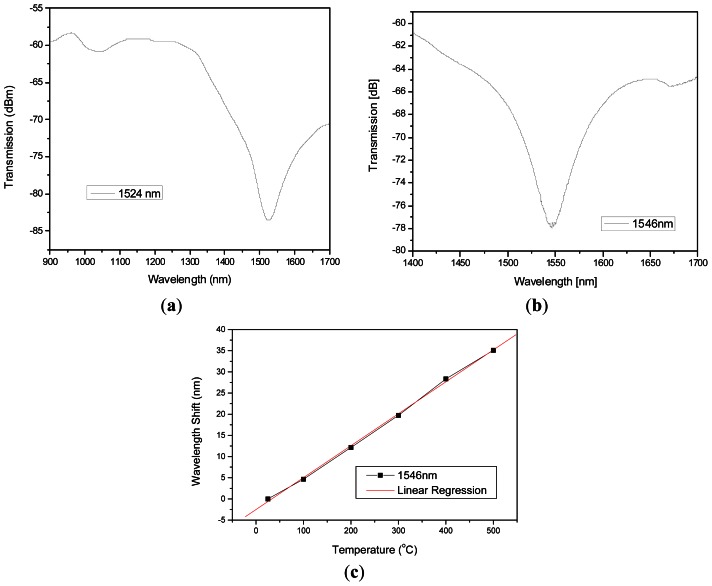
(**a**) Transmission spectra of 1,524 nm F-LPFG, (**b**) Transmission spectra of 1,546 nm F-LPFG, (**c**) Linear regression for 1,546 nm F-LPFG.

The spectra recorded with the OSA; in log scale, shows a common linear wavelength shift response as temperature increases for F-LPFGs at 1,524 and 1,546 nm central dip wavelengths, Figure 4.

**Figure 5.**
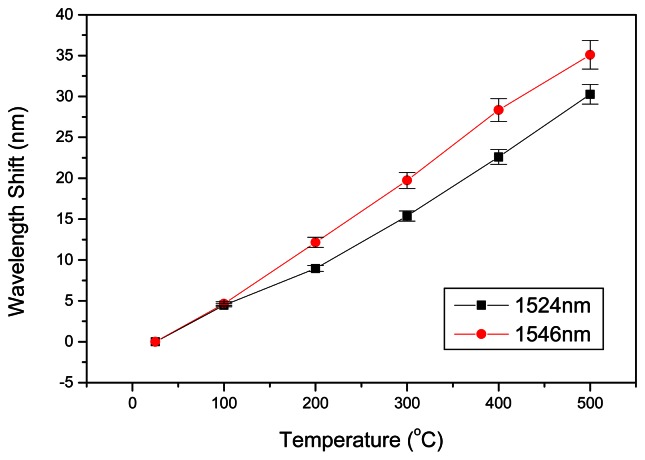
Transmission spectra of both F-LPFG with variations of maximum of 5%.

The sensitivity achieved was ∼72 pm/°C, for the case when the experimental temperature rate was 0.17 °C/s, which is a greater value than the ones obtained in previous works with DS F-LPFG at1,550 nm (where the fiber was fattened at positions separated by ∼25 μm) [17]. The transmission spectrum is similar to almost all LPFGs. The attenuation band shifts to longer wavelengths while the depth of the band is attenuated as the temperature rises. The evolution of the isolation loss with temperature in a range from (25–500 °C) is about −1.5 dBm. These effects have been observed and reported in many literature or experiments with LPFGs. The repeatability was measured several times. The temperature was increased to the limit of 500 °C and then to low temperature. The fiber was exposed 10 min at increments of temperature of 100 °C. There were small variations during the characterizations which are illustrated in Figure 5. The overall temperature response time measured was within 3 s for this sensor.

If we wanted to build an electronic device to read the movement of the band, so it would be more complicated to detect the central wavelength. For this reason the change of logarithmic to the linear regime to which, instead of reading the displacement, power changes can be read in intensity as the resonance band changes with temperature.

**Figure 6.**
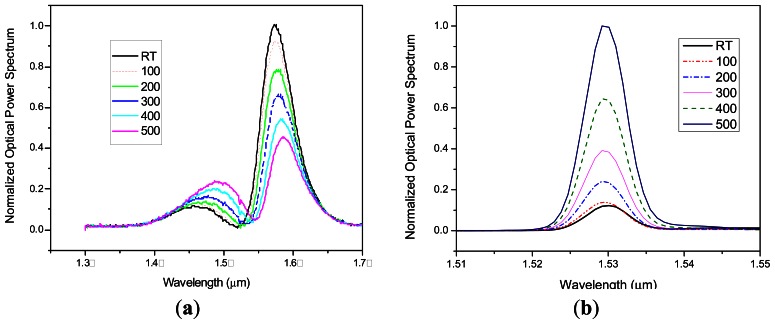
(**a**) Power spectrum of a 1,524 nm F-LPFG, (**b**) Power spectrum of a 1,546 nm F-LPFG.

Measurements of light power variations of F-LPFGs with temperature could be registered with the OSA in the linear scale. The fluorescence spectrum of erbium in the wavelength range underneath ∼1,532 nm has a positive slope and wavelengths over 1,540 nm has a negative slope [19], for this reason the increase of the wavelength of the F-LPFG that is centered at 1,546 nm when increasing temperature caused an increase on the power measurements and the ones centered at 1,524 nm caused a decrease on the power measurements. Transmission spectra for F-LPFG at 1,524 nm and 1,546 nm with an erbium source are shown in Figure 6.

**Figure 7.**
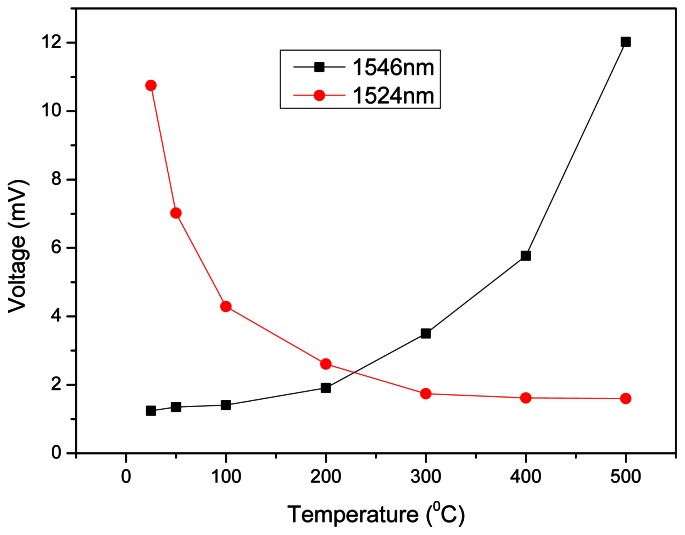
Raise and fall of voltage due to temperature changes of 1,524 nm and 1,550 nm F-LPFGs.

As part of the power spectrum is filtered with the 1,524 nm F-LPFG, it keeps enough power which decreases as the temperature increases (Figure 6(a)). This is the effect that occurs when placing a LPFG near the left end of the fluorescence spectrum. The F-LPFG at central wavelength of 1,546 nm immediately attenuates the power spectrum and when temperature increases, from room temperature to 500 °C, produces the increment of power as depicted in the Figure 6(b). These measurements were made with a maximum source power of 50 mW. After observing the power behavior of the F-LPFG with the erbium source, the goal is to measure the F-LPFG output as a variable easy to read by any electronic device as an oscilloscope or electronic homemade board that can help to characterizein volts/°C and achieve a very compact temperature sensor. Figure 7 shows these steady changes as temperature varies every ±100 °C. The highest sensitivity registered in volts is 0.05 mV/°C.The curves in Figure 7 are not linear. This effect can be avoided using wider and flattened spectral sources. Some candidates are photoluminescent diodes with a high emission intensity, Erbium doped fibers with wide flattened spectrum can also be used or a supercontinuum wide spectrum white light source.

One of the main advantages of the current sensor is that in contrast with other long period fiber gratings fabricated by electric arc, CO_2_ or others, this device is totally insensitive to changes in the external refractive index and has very low sensitivity to bending compared with other long period gratings where the length may be in excess of 1 cm [6,20]. In the case of LPGs fabricated by UV, the application of high temperature may erase the gratings in most cases. Hence for this particular application the F-LPFG has a clear advantage with respect to UV LPFG of similar lengths, and with respect to other arc-induced LPFG has the advantage of lower sensitivity to external factors like refractive index and bending, although it has temperature sensitivity in the same order. Furthermore, the OSA can be replaced by a small power meter or any compact electronic device capable to convert light into voltage or data which can enormously reduce the size of a complete optical sensor.The optical device results very compact due to the compact grating and the compact electronic device.

## 5. Conclusions

Compact fattened DS-LPFGs can be fabricated with lengths of ∼2 mm with a dynamic wavelength range ∼36 nm for a temperature range from 25 to 550 °C. The measured sensitivity of 72 pm/°C improves the sensing devices fabricated with F-LPFG. The advantage of getting a LPFG with a very broad spectrum is that, with steep spectra sources, sensors whose behavior will depend on the position of the central dip wavelengths, can be deployed. In this way, it is easier to obtain the signal sensitivity (*i.e.*, voltage) which in this case turned out to be 0.05 mV/°C. The small sensor based on these shortF-LPFGs has potential applications where smaller devices are required without loss of performance and reliability as in ovens, medicine, bio-sensing, optical communications, and actual opto-mechatronics systems in industry.
